# Pilot Randomized Controlled Trial of the WHO Caregiver Skills Training in Public Health Services in Italy

**DOI:** 10.1007/s10803-021-05297-x

**Published:** 2021-10-22

**Authors:** Erica Salomone, Michele Settanni, Helen McConachie, Katharine Suma, Federica Ferrara, Giulia Foletti, Arianna Salandin, WHO CST Team, Felicity L. Brown, Laura Pacione, Stephanie Shire, Chiara Servili, Lauren B. Adamson

**Affiliations:** 1 Department of Psychology, University of Milano-Bicocca, Piazza dell’Ateneo Nuovo 1, 20126 Milan, Italy; 2 Department of Mental Health and Substance Use, World Health Organization, Geneva, Switzerland; 3 Department of Psychology, University of Turin, Turin, Italy; 4 Population Health Sciences Institute, Newcastle University, Newcastle upon Tyne, UK; 5 Department of Human Development and Family Science, University of Georgia, Athens, GA USA; 6 Department of Psychology, Georgia State University, Atlanta, Geogia USA

**Keywords:** Caregiver skills training, Parenting education, Autism spectrum disorders, Parent–Child relations, Public health, Randomized controlled trial

## Abstract

Parents of children with ASD (N = 86; mean age 44.8 months; 67 boys) were randomized to either WHO Caregiver Skills Training (CST) delivered in public health settings in Italy or enhanced treatment-as-usual. Primary blinded outcomes were 3-months post-intervention change scores of autism severity and engagement during caregiver-child interaction. CST was highly acceptable to caregivers and feasibly delivered by trained local clinicians. Intention-to-treat analysis showed a large and significant effect on parent skills supporting joint engagement and a smaller significant effect on flow of interaction. Expected changes in child autism severity and joint engagement did not meet statistical significance. Analysis of secondary outcomes showed a significant effect on parenting stress, self-efficacy, and child gestures. Strategies to improve the effectiveness of CST are discussed.

## Introduction

Joint engagement experiences with partners and shared activities are fundamental for the emergence of communication skills, the mutual regulation of affect, the development of problem solving abilities and the sharing of cultural meaning (Adamson et al., 2009). Children with autism spectrum disorder (ASD) spend significantly less time jointly engaged with communicative partners than typically developing children and children with developmental delay (Adamson et al., 2009), which likely has significant effects on their development (Bottema‐Beutel, 2016; Mundy et al., 1990). The reduced exposure to joint engagement states occurs as children with ASD display a reduced ability in both responding to joint attention and initiating joint attention (Mundy et al., 2007): children with ASD are more frequently unaware of or reject bids for joint engagement (Adamson et al., 2001) and display fewer communicative acts to share interests in objects or events (Landa et al., 2013; Watson et al., 2013). These behavioral patterns disrupt the expectations and strategies adults intuitively use to successfully sustain engagement, which often makes parents of children with ASD who have not received intervention less effective in establishing joint engagement. Indeed, prior to receiving intervention, parents of children with ASD display a lower ability to ‘scaffold’ (Bruner, 1985), or support, shared activities and to ‘follow-in’ (Tomasello & Farrar, 1986), or reference, the child’s focus of attention in play interactions, compared to parents of typically developing children or children with developmental delay (Adamson et al., 2019). The effects on parental psychological wellbeing of such repeated perceived ‘failures’ at attempts to engage the child are not negligible, as they may lead to self-doubt and reduced confidence in the ability to parent effectively. Lower parental self-efficacy, in turn, is associated with fatigue and lowered wellbeing which may further exacerbate parenting difficulties (Giallo et al., 2013).

To protect caregiver wellbeing and improve long-term child outcomes, caregivers of children with ASD therefore need sensitive and specific support to develop skills to create and sustain joint engagement experiences within every-day interactions with their children. There is evidence that parent-mediated interventions can change the way parents interact with their children (Oono et al., 2013). However, the majority of this evidence is derived from efficacy trials conducted within controlled university settings and with highly specialized clinical personnel (Hardan et al., 2015; Kasari et al., 2010; Pickles et al., 2016; Wetherby et al., 2014), whereas when these models are implemented in community settings the effectiveness is considerably reduced. A meta-analysis of community-based early intervention for cognitive, communication, social, and adaptive behavior outcomes found small effect sizes (0.21- 0.32) for children with ASD (Nahmias et al., 2019), while previous meta-analyses of university-based clinical trials report substantially higher effect sizes (0.42 – 0.76) for the same domains (Reichow, 2012).

Primary challenges of implementing parent-mediated interventions in community settings include: (a) the threats to fidelity and integrity of the intervention due to a reduced expertise of interventionists, (b) the heterogeneity of participants by socioeconomic status (SES) and clinical characteristics, which increases the variability in the sample and (c) the limited control over the design (such as amount and quality of usual care). However challenging, investigating these models under real-world conditions should nonetheless be pursued as it can accelerate the process of bridging the gap between research and clinical practice (Weisz et al., 2015). As the global burden of disease for neurodevelopmental disorders is predicted to gradually increase (Whiteford et al., 2013), there is indeed an urgent need to increase access to evidence-based intervention through public health services. This applies not only to low-and-middle income countries (LMIC), where notoriously the majority of children with developmental disorders do not have access to care, but also to high-income countries (HIC), where family characteristics such as race, ethnicity, and SES contribute to service disparities (Smith et al., 2020).

In response to the need for an open-access, evidence-based, feasible in low-resource contexts caregiver training program, the World Health Organization (WHO) promoted the development of a novel program for developmental disorders, the ‘WHO Caregiver Skills Training for Families of Children with Developmental Delays and Disabilities’ (hereafter: CST). The CST program was developed as part of the Mental Health Gap Action Programme (mhGAP), an initiative aiming to bridge the ‘treatment gap’ for priority mental, neurological and substance use conditions (Saxena, 2016). The development of the CST program, which is founded on principles of developmental science, social communication interventions, applied behavior analysis, positive parenting, and self-care methods, was informed by evidence reviews, meta-analyses and consultations with experts and users (Salomone et al., 2019). At the core of the intervention methodology in CST is the shaping of every-day activities into shared caregiver and child routines so that children have regular joint engagement experiences that provide opportunities for the development of new skills. Through modelling, coaching and group discussions, caregivers are shown strategies to improve their ability to scaffold the child’s activities, follow the child’s lead and use positive affect to establish and maintain routines.

The present study examined the acceptability, feasibility and indicators of effectiveness of community-implementation of the CST in Northern Italy. Italy is a HIC where the provision of evidence-based interventions through public Child Neuropsychiatry services is free, but with several areas significantly under-served. An Italian National Institute of Health survey reported that 50% of public child neuropsychiatry services do not offer any kind of ASD-specific interventions (Borgi et al., 2019), confirming earlier parent-report evidence that publicly provided ‘treatment as usual’ (TAU) for ASD mostly consists of either speech and language therapy or non-specific occupational therapy (‘psychomotor therapy’), with two-thirds of families not accessing parent training/education (Salomone et al., 2016). Personnel shortages and limited access to specific professional training are thought to be among the underlying reasons for the current state of public intervention provision (Borgi et al., 2019). To examine whether the CST could address these limitations, we first undertook a pre-pilot implementation of CST in Northern Italy. This pre-pilot was the first ‘test-run’ of the CST program globally. It showed good feasibility and acceptability of key intervention components and delivery methods and informed, together with early implementation data from other sites, such as Ethiopia (Tekola et al., 2020), the global field testing initiative (Salomone et al., 2019). We then conducted an effectiveness-implementation hybrid pilot randomized controlled trial (RCT) in public Child Neuropsychiatry services of the Piedmont region in Northern Italy. Reported here are the clinical outcomes of the pilot RCT; the formative adaptation process and acceptability and feasibility data from the trial are reported elsewhere (Salomone et al., 2021).

## Methods

### Design

The design of the pilot implementation was a two-arm, single (assessor)-blinded RCT of CST against enhanced treatment as usual (eTAU: one psychoeducation session in addition to TAU). Data were collected at baseline, immediately post-intervention (3 months post-baseline) and 3 months post-intervention.

### Participants

Children (n = 86) were recruited to the study as per the following inclusion criteria: (a) child’s age between 24 and 60 months; (b) clinical diagnosis of ASD by ICD-10 criteria obtained using a combination of semi-structured observations, parent interviews and school reports by local clinicians and confirmed by research assessments. Exclusion criteria were: (a) level of spoken Italian in the caregiver insufficient to fully participate in the intervention; (b) psychiatric conditions in either of the parents as reported in the clinical notes. Children were not excluded on the basis of level of intellectual disability or any co-occurring conditions. Baseline characteristics are reported in Table 1.

**Table 1:** Baseline characteristics of participants

		eTAU (n = 43)		WHO CST (n = 43)		
	*N*	*n (%)*	*M (SD)*	*n (%)*	*M (SD)*	*p*
*Child*						
Male	86	34 (79.1)		33 (76.7)		.500
Age in months	86		44.21 (9.01)		45.56 (10.06)	.514
Time since diagnosis	81		13.86 (10.48)		14.38 (9.96)	.603
Cognitive ability (Griffiths III GD AE)	86		22.84 (6.98)		23.84 (6.93)	.507
Autism severity (ADOS-2)	85					
Total CSS			7.00 (2.00)		6.83 (2.34)	.725
SA CSS			6.93 (2.18)		6.95 (2.18)	.963
RRB CSS			8.47 (1.55)		7.95 (2.06)	.197
Level of language (ADOS-2 Item A1)	86					
Non-verbal		13 (30.2)		13 (30.2)		.839
Single words		24 (55.8)		21 (48.8)		
Two-word utterances		3 (6.9)		4 (9.3)		
Phrase speech		3 (6.9)		5 (11.6)		
Current use of medication	86					
Sleep medication		5 (11.6)		10 (23.3)		.155
Antiepileptic medication		1 (2.3)		1 (2.3)		1.0
*Primary caregiver*						
Mother	86	37 (88.1)		30 (69.7)		.069
Age	84		36.6 (5.51)	36.88 (5.6)		.815
Non-Italian nationality	86	12 (27.9)		14 (32.6)		.621
Educational level	84					
Elementary/middle school		11 (26.2)		9 (21.4)		.687
High school diploma		24 (57.1)		23 (54.8)		
Degree and post-degree		7 (16.7)		10 (23.8)		

*Griffiths III GD AE* Griffiths III General Development age equivalents, *ADOS-2* Total CSS, SA CSS, RRB CSS Autism Diagnostic Observation Schedule Second Edition Composite Severity Scores for Total, Social Affect and Restricted and Repetitive Behaviors, *ADOS-2 Item A1* Autism Diagnostic Observation Schedule Second Edition, Item A1 'Overall level of non-echoed spoken language', across modules

### Baseline Measures

#### Autism Severity

The diagnosis was confirmed with the Autism Diagnostic Observation Schedule Second Edition (ADOS-2, Lord et al., 2012), administered by two chartered clinical psychologists (FF, AS), who met 80% reliability criteria with the first author, a chartered clinical psychologist and accredited ADOS-2 Trainer. All children were above the cutoff scores on the Overall Total for ASD.

#### Cognitive Skills

The Italian version of the Griffiths Scales of Child Development, Third Edition (Griffiths III, Green et al., 2016; Lanfranchi et al., 2017) was administered. The age equivalents scores for the General Development composite were used in the analysis.

#### Receipt of Usual Care

Access to usual care was measured with a comprehensive semi-structured interview derived from a questionnaire previously used in European samples to describe receipt of treatment as usual (Salomone et al., 2016). The questions probed for child-directed services, support at school, parenting programs or counselling received in the six months prior to the trial. The interview was repeated immediately post-intervention and 3 months post-intervention to record access to care during the trial.

### Randomization and Blinding

A total of 88 children were referred to the study through local child neuropsychiatry services. Two children not meeting the age criteria were subsequently excluded. All 86 remaining children were enrolled; informed written consent was obtained from both parents/guardians. Following baseline ascertainment, participants, identified by sequentially assigned identification numbers, were randomly assigned by an independent statistician to either CST (n = 43) or eTAU (n = 43) on a 1:1 allocation ratio using stratified randomization by age (below 42 months and 42 months and above) and autism severity (ADOS-2 Comparative Severity Score: minimal/low and moderate/high algorithm categorizations). These characteristics were selected as factors that may influence the treatment response. Allocation was conveyed by email to the site coordinator who relayed it to the intervention team. The research and intervention teams used separate office facilities. Research assistants, who were masked to treatment allocation, rated baseline, immediate post-intervention and 3-months post intervention measures from anonymized video-recordings without indication of arm or timepoint.

### Intervention

The 12-sessions CST intervention program includes 3 home visits and 9 group sessions training caregivers via adult-learning techniques (Salomone et al., 2019). The first Home Visit is aimed at goal setting and is conducted before the first group session, the second one focuses on coaching and occurs at the mid-point of the program; the third home visit delivers coaching and support for independent practice and occurs after the last group session. The group sessions cover the following topics: getting and keeping children engaged (Sessions 1–2); building home and play routines (Session 3); understanding and promoting communication (Sessions 4–5); preventing and reducing challenging behavior (Sessions 6–7); promoting daily living skills (Session 8); caregiver wellbeing and problem solving (Session 9). Each session includes a wellness activity (breathing exercise), a review of the previous session and of home practice, a discussion of a caregiver story (illustrated clinical vignette), the presentation of new content with the aid of visuals, the demonstration (modelling) of intervention strategies, the caregiver role play and the guided plan for home practice. Caregivers are expected to practice independently at home with the intervention strategies; the home practice is reviewed during the group sessions.

The CST program was delivered per manual in six public child neuropsychiatry services of the Italian National Health System by six pairs of local clinicians. The clinicians received the standard 5-day training course including presentations, role plays and practice with volunteer families and four post-training supervised practice sessions with families delivered by a WHO CST Team member ES; all interventionists met post-training CST competency criteria. CST group size varied from 5 to 8 families. Participation in the program was open to 1–2 caregivers per family; data were collected on a target caregiver/child dyad designated by the family at baseline. The group sessions lasted 2.5–3 h and were held at the local child neuropsychiatry facilities; children were not present at the sessions. The home visits were delivered at participants’ homes and lasted 1.5 h. Clinicians’ intervention fidelity was checked during the program delivery and was acceptable both for the integrity of group sessions delivery and the fidelity of implementation of CST strategies in direct interaction with the child during the home visits. (For further detail on the intervention procedure, see Salomone et al., 2021).

### Feasibility and Acceptability

After each session, caregivers and interventionists completed feasibility and acceptability measures. Focus groups and interviews were conducted with interventionists and caregivers immediately post intervention. For the full set of measures, including qualitative data from focus groups, see Salomone et al. (2021). ‘Unsatisfactory’ levels were set at ≤ 3 on 1–5 scales.

### Outcomes

Baseline data (T1) were available for all primary and secondary outcomes. Considering the nature of the intervention, whose theory of change assumes that effects on child outcomes are mediated by the uptake of improved interaction strategies in the caregiver, we postulated that effects could be detectable only several weeks post intervention to allow for independent practice of strategies. For this reason, all primary and secondary outcomes were measured 3 months after the last intervention session, i.e. the final Home Visit (hereafter: T3, or ‘3 months post-intervention’). Since we expected that some early effects could be detectable immediately after the last intervention session (T2), data were also collected at that timepoint on all child and caregiver primary outcomes and on all caregiver, but not child, secondary outcomes.

### Primary Outcome Measures

#### Autism Symptom Severity

The Brief Observation of Social Communication Change (BOSCC; Grzadzinski et al., 2016) is a measure of change in social communication behaviors developed based on ADOS-2 codes. The tool is under development; Version July 27, 2017 was used in this study. When applied to caregiver/child interaction, the BOSCC consists of 15 items rating the child’s behavior: nine items consider social-communication skills (such as eye contact, gestures, social overtures), three items rate restricted and repetitive behaviors (including sensory interests, mannerisms and stereotyped behaviors) and the last three items describe behaviors not specifically associated with, but frequently occurring in, ASD (hyperactive, disruptive, and anxious behaviors). All items are coded on a 6-point scale (0 – abnormality is not present to 5 – abnormality is present and may significantly impair functioning) with the aid of a decision tree. Averaged scores were obtained for two 5-min segments that were scored separately.

#### Dyadic Engagement

**Fig. 1:**
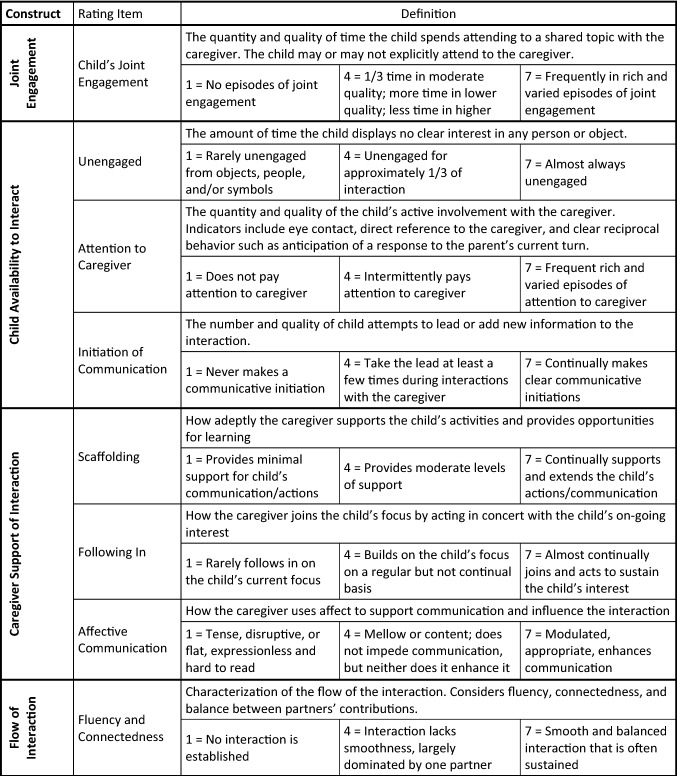
Joint Engagement Rating Inventory (JERI) constructs and rating items

The Joint Engagement Rating Inventory (JERI; Adamson et al., 2020) is a measure designed to characterize various aspects of joint engagement that occur as caregivers interact with typically developing toddlers between 18 and 30 months old and young children with developmental delays, including those diagnosed with ASD. The current version of the JERI contains 32 rating items that have been constructed as researchers have adapted the original set of rating items (Adamson et al., 2012) to suit specific studies; as recommended in the manual, the current study selected a subset of items germane to the research questions. Eight items were used: two engagement items – child joint engagement and child unengaged; two child behavioral items – initiation of communication, attention to caregiver; three caregiver behavioral items – scaffolding, following in on the child’s focus, affective communication; and one dyadic interaction item – fluency and connectedness. Items are scored on a 7-point rating scale (1 – feature is minimally present, 7 – feature is highly present). See Fig. 1. Four variables were derived from the rating items to measure the constructs of *Joint engagement* (one item: Child's Joint Engagement); *Child availability to interact* (three items: reversed scored Unengaged, Attention to Caregiver, Initiation of Communication); *Parent support of interaction* (three items: Scaffolding, Following In, Affective Communication): and *Flow of interaction* (one item: Fluency and Connectedness).

#### Procedure to Obtain the Primary Outcome Measures

The primary outcome measures were derived from a free play caregiver-child interaction with a standard toy kit suitable for a range of developmental play levels videorecorded at baseline, immediately post-intervention and 3 months post intervention, at the child’s home. Parents were instructed to play as they would usually do. Ten consecutive minutes of an approximately 12-min interaction were rated to obtain the primary outcome measures. Rating begun when the dyad had settled and either the parent or child engaged with an object. Rating procedures were applied as per the manual for each measure by two observers (FF and GF), both clinical psychologists fluent in Italian and experienced in clinical work with young children with ASD. Raters achieved a high level of agreement with the master trainer (ES) before rating the video corpus. Prior to data coding, the master trainer trained the two raters until the recommended reliability standards were met; throughout data collection the raters met with the master trainer to discuss ongoing reliability. Raters were blind to the study’s hypotheses, group allocation and time point of the assessment. The video corpus was rated with the BOSCC first, and subsequently with the JERI.

#### Reliability of the Primary Outcome Measures

A total of 256 interactions were rated. To check agreement, 22% of the corpus of each observer was independently rated by a second trained observer; observers did not know which of their sessions were double coded. The inter-rater reliability on the double-coded videos was excellent both for the BOSCC on the Total score (intraclass correlation coefficients, ICC = 0.92) and for the JERI items (range of weighted Kappas (Cohen, 1968): 0.89–1.0).

### Secondary Outcomes

#### Child Vocabulary and Gestures

Parents completed the Italian version (Caselli et al., 2007) of the MacArthur-Bates Communicative Development Inventories (MCDI, Fenson et al., 2007). The MCDI shows very high concurrent validity with direct assessments (Nordahl-Hansen et al., 2014) and the inter-rater reliability of parent and teacher ratings is excellent (Nordahl-Hansen et al., 2013). Total endorsed receptive and expressive words raw counts (maximum possible score for each total: 408) and gestures raw counts (maximum possible score: 12) were used in the analysis.

#### Child Adaptive Behavior

Parents were interviewed with the Italian version (Balboni et al., 2016) of the Vineland II (VABS, Sparrow et al., 2005), a semi-structured interview that rates the child’s current level of functioning across the domains of Communication, Daily Living and Socialization. Age-normed Standard Scores (M = 100; SD = 15) for the Adaptive Behavior Composite (ABC) were used in analyses.

#### Parenting Self-Efficacy

Parenting self-efficacy was assessed with a general measure of parenting satisfaction and efficacy, the 17-item self-report Parenting Sense of Competence Scale (PSOC, Johnston & Mash, 1989) and the Caregiver Self-efficacy Questionnaire, (CSQ, included in the WHO Caregiver Knowledge and Skills Test, WHO, unpublished), a 13-item 5-point scale measure of parenting self-efficacy applied to domains relevant for parenting a child with developmental delay (e.g. promoting skills development, inclusion, coping with challenging behavior). The PSOC has good internal reliability (α = 0.75–0.88, Johnston & Mash, 1989; Lovejoy et al., 1997), but uncertain factor structure (Gilmore & Cuskelly, 2009). The Total score was used in analysis for both measures; internal reliability (α) in this sample was excellent for both the PSOC (0.81) and the CSQ (0.88).

#### Parental Stress

Parental stress was measured with an autism-specific questionnaire, the Autism Parent Stress Index (APSI, Silva & Schalock, 2012). The APSI is a 13 item self-report questionnaire examining parenting stress related to a child’s ASD core deficits, behavioral symptoms, and co-morbid physical symptoms. It showed adequate internal reliability in parents of children with ASD (α = 0.67 − 0.83), good test–retest reliability (r = 0.88), good discriminant validity among parents of children with ASD, DD, and typically developing children (Silva & Schalock, 2012). The α in this sample was excellent (0.87).

### Statistical Analyses

To identify possible significant differences between groups at baseline, we conducted independent t-tests on continuous variables and chi-square analyses on categorical or nominal variables at baseline.

Univariate effects of group membership on 3-months post-intervention change scores of the primary outcomes were assessed using between-subjects ANCOVA, adjusting for baseline measures. Similarly, as a secondary analysis we then analyzed the changes in the primary outcomes as observed immediately post-intervention with ANCOVA analysis of the change scores, using the baseline levels as covariates.

The same analyses were repeated for the caregiver secondary outcomes, using two ANCOVA models to analyze, respectively, the change scores calculated 3-months post-intervention and immediately post-intervention controlling for outcome values at baseline. Child secondary outcomes were only examined with ANCOVA analysis of change scores 3-months post-intervention since, as described above, for those measures data immediately post-intervention were not collected.

We assessed effect size using partial eta squared (η_p_^2^), and interpreted it following the guidelines of Cohen (1988): η_p_^2^ were interpreted as small (η_p_^2^ = 0.01), medium (η_p_^2^ = 0.06), and large (η_p_^2^ = 0.14) effects. All models were estimated using intention-to-treat (ITT) analysis. We used multiple imputation with chained equations to impute missing outcome data in the analysis of all outcomes. The ITT analysis was conducted for all available data. The general significance level was set to 0.05. All descriptive computations were conducted using SPSS 27 (IBM Corp., Armonk, NY, USA), whereas the imputation and inference (ANCOVA) were carried out using R 4.04 (R Core Team, 2017) with the packages mice (Buuren & Groothuis-Oudshoorn, 2010) and miceadds (Robitzsch & Grund, 2021). Missing data was multiply imputed using the predictive mean matching method.

## Results

**Fig. 2:**
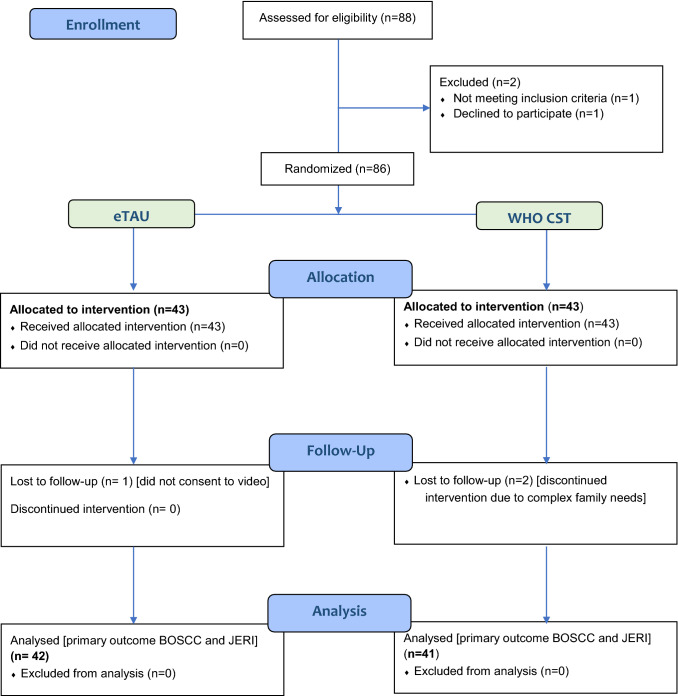
CONSORT Flow chart

Figure 2 shows the CONSORT diagram of participant flow through the study. A total of 3/86 (3.5%) participants (2 from CST and 1 from eTAU) were lost from follow-up; primary outcome data (BOSCC and JERI ratings) were available for 83 (96%) subjects (41 from the treatment arm and 42 from the control arm).

### Baseline Characteristics

The two groups did not differ on any of the baseline characteristics (Table 1). Baseline levels of outcome measures are reported in Table 2.

### Feasibility and acceptability

The intervention delivery integrity ratings (group sessions) ranged from 82 to 97% across sites. Fidelity of implementation of intervention strategies in interaction with children (home visits) was 75.99% (3.69%); average fidelity per site ranged from 73.17% to 80.15%. Among actively enrolled intervention parents (n = 39), 84% completed at least 75% of the intervention. Caregiver-rated acceptability (comprehensibility, relevance, alignment with values) was above satisfactory levels in 97% of ratings across sessions (n = 215). Interventionist ratings (n = 101) of acceptability to caregivers and feasibility of delivery were above satisfactory levels in, respectively, 97% and 86% of ratings across sessions and dimensions (perceived relevance, acceptability, agreement and participation; complexity and amount of contents, preparedness to deliver). For a break-down of acceptability and feasibility ratings per session, see Salomone et al. (2021).

### Receipt of Usual Care

Access to usual care was not restricted for either group. The two arms did not differ on hours of child-directed services (*p* = 0.205), support at school (*p* = 0.383), parenting programs or psycho-educational counselling (*p* = 0.197) received during the trial.

### Effects on Primary Outcomes

One-way ANCOVAs were conducted to analyze the effects of the intervention on the primary outcome variables, i.e. BOSCC and JERI change scores between baseline and 3-months post-intervention, while controlling for the respective baseline levels (Table 2).

**Table 2:** Effects of intervention on primary outcomes 3 months post intervention (T3)

	eTAU				WHO CST						
	Baseline		3-months post-intervention		Baseline		3-months post-intervention		Mean change difference*		
	Mean	SD	Mean	SD	Mean	SD	Mean	SD	Mean [95% CI]	*η*_*p*_^*2*^	*p*
BOSCC Total score	31.49	11.33	29.70	12.28	27.35	10.93	25.39	10.75	-0.71 [-3.45, 2.02]	< 0.001	.99
JERI Joint engagement	3.02	2.14	3.52	2.03	3.44	2.07	4.35	2.02	0.53 [-0.07, 1.14]	0.03	.19
JERI Child availability	11.95	4.74	12.25	4.80	12.65	4.43	13.51	4.31	0.69 [-0.43, 1.81]	0.01	.56
JERI Parent support of interaction	11.16	3.99	11.57	4.73	11.44	4.56	14.84	4.29	3.08 [1.66, 4.49]	0.18	< .001
JERI Flow of interaction	3.58	1.62	3.93	1.56	3.86	1.67	4.66	1.59	0.54 [0.07, 1.02]	0.05	.03

ITT analysis, ANCOVA: means from baseline and 3 months post intervention, mean change difference and effect size. p-values are from the ANCOVA based on multiple imputation. A p-value < 0.05 was considered statistically significant

*Mean change difference 3-months post-intervention controlling for outcome measured at baseline

*ANCOVA* analysis of covariance; *BOSCC* Brief Observation of Social Communication Change, *JERI* Joint Engagement Rating Inventory

In regard to the BOSCC, we analyzed the change scores for the *Total score*. Controlling for baseline levels, the difference in change score between the two groups was non-significant, with the effect size value very close to 0 (< 0.001), indicating no significant effect of the intervention.

With respect to the JERI, we conducted analyses on the following subscales: *Joint engagement*, *Child availability to interact*, *Parent support of interaction*, *Flow of interaction*. For *Joint engagement* the effect of the intervention was not significant, but the size of the effect is worthy to note (η_p_^2^ = 0.03), given that it is in the expected direction and that it is reasonable to expect that with a larger sample size the effect would have reached the statistical significance. A significant effect emerged for *Flow of interaction*. Individuals in the experimental group had a significantly greater increase in their score on this variable than those in the control group, η_p_^2^ = 0.05. For the *Parent support of interaction* subscale the effect of the intervention was large and significant (η_p_^2^ = 0.18). Finally, with respect to the *Child availability to interact* subscale, no significant effect emerged.

In order to study the early effects of the intervention we conducted the same analyses on change scores immediately after the end of the treatment (Table 3). The intervention had no significant effect on the study outcomes except for the JERI *Parent support of interaction*, which significantly increased in the treatment group with respect to the control condition (η_p_^2^ = 0.06). It is worth noting that the effect on *Parent support of interaction*, which was the strongest intervention effect 3 months post-intervention (η_p_^2^ = 0.18), was already present immediately after the treatment, although with a lower effect size (η_p_^2^ = 0.06).

**Table 3:** Effects of intervention on primary outcomes immediately post intervention (T2)

	Post-intervention						
	eTAU		WHO CST		Mean change difference*		
	Mean	SD	Mean	SD	Mean [95% CI]	*η*_*p*_^*2*^	*p*
BOSCC Total score	30.24	11.31	27.20	10.73	0.30 [−2.48, 3.07]	0.01	.45
JERI Joint engagement	3.48	1.97	3.91	2.22	0.13 [−0.53, 0.8]	0.00	.88
JERI Child availability	11.97	3.91	13.14	4.12	0.67 [−0.29, 1.63]	0.01	.39
JERI Parent support of interaction	11.77	4.11	13.97	4.74	2.04 [0.46, 3.61]	0.06	.03
JERI Flow of interaction	3.89	1.54	4.16	1.64	0.10 [−0.42, 0.62]	0.00	.84

ITT analysis, ANCOVA: means from immediate post-intervention, mean change difference and effect size. p-values are from the ANCOVA based on multiple imputation. A p-value < 0.05 was considered statistically significant

*Mean change difference from baseline to immediately post-intervention controlling for outcome measured at baseline

*ANCOVA* analysis of covariance, *BOSCC* Brief Observation of Social Communication Change, *JERI* Joint Engagement Rating Inventory

### Effects on Secondary Outcomes

Table 4 shows the results of the ANCOVA analysis conducted on the 3-months post-intervention change scores of secondary outcomes. Significant effects of the intervention emerged with regard to the increase in ASD-specific parental self-efficacy, measured by the CSQ, and the decrease in the level of parental stress measured by the APSI (η_p_^2^ = 0.06 and η_p_^2^ = 0.05, respectively). Furthermore, the intervention was effective in significantly increasing the number of child spontaneous gestures, as measured by the MCDI, η_p_^2^ = 0.05. No other statistically significant effects emerged.

**Table 4:** Effects of intervention on secondary outcomes 3 months post intervention (T3)

	eTAU				WHO CST						
	Baseline		3-months post-intervention		Baseline		3-months post-intervention		Mean change difference*		
	Mean	SD	Mean	SD	Mean	SD	Mean	SD	Mean [95% CI]	*η*_*p*_^*2*^	*p*
*Child*											
MCDI Gestures	7.15	3.62	7.96	3.53	7.26	9.12	8.85	8.75	0.82 [0.05, 1.61]	.05	.04
MCDI Expressive words	71.26	116.96	130.76	154.02	107.14	142.99	183.20	159.48	17.4 [-21.79, 56.58]	.009	.38
MCDI Receptive words	197,02	123,90	253,27	121,04	207,24	129,51	274,00	114,87	12,44 [-12.1, 36.98]	.01	.32
VABS ABC	55.98	16.90	57.56	19.35	56.98	15.80	60.02	17.04	1.77 [-4.3, 7.83]	.004	.57
*Primary caregiver*											
PSOC Total Score	69.00	12.18	67.18	10.93	66.05	10.43	67.19	11.37	2.04 [-1.69, 5.78]	.02	.29
CSQ Total Score	47.79	7.14	47.54	7.48	47.18	7.98	50.15	7.79	2.97 [0.36, 5.59]	.06	.03
APSI Total Score	30.66	8.66	30.91	9.35	29.10	8.84	27.26	6.07	-2.72 [-5.2, -0.23]	.05	.03

ITT analysis, ANCOVA: means from baseline and follow-up, mean change difference and effect size. p-values are from the ANCOVA based on multiple imputation. A p-value < .05 was considered statistically significant

*Mean change difference 3 months post-intervention controlling for outcome measured at baseline

*ANCOVA* analysis of covariance, *MCDI* MacArthur-Bates Communicative Development Inventories, *VABS CSS* Vineland II Adaptive Behaviour Composite standard score, *PSOC* Parenting Sense of Competence Scale, Caregiver Self-efficacy Questionnaire, *APSI* Autism Parent Stress Index

With respect to the analyses conducted on the secondary outcomes immediately post-intervention, shown in Table 5, only the effect of the intervention on the APSI score was significant, with η^2^ = 0.05, again in the expected direction of a reduction in parental stress.

**Table 5:** Effects of intervention on secondary outcomes immediately post intervention (T2)

	Post intervention						
	eTAU		WHO CST		Mean change difference*		
	Mean	SD	Mean	SD	Mean [95% CI]	*η*_*p*_^*2*^	*p*
*Primary caregiver*							
PSOC Total Score	67.74	1.672	67.08	9.55	1.02 [-2.64, 4.68]	.01	.59
CSQ Total Score	48.30	1.236	50.69	6.97	2.71 [0.00, 5.43]	.04	.05
APSI Total Score	30.58	1.431	26.87	7.10	-2.75 [-5.47, -0.02]	.05	.048

ITT analysis, ANCOVA: means from immediately post intervention, mean change difference and effect size. p-values are from the ANCOVA based on multiple imputation. A p-value < 0.05 was considered statistically significant

*Mean change difference immediately post intervention controlling for outcome measured at baseline

*ANCOVA* analysis of covariance, *PSOC* Parenting Sense of Competence Scale; Caregiver Self-efficacy Questionnaire, *APSI* Autism Parent Stress Index

### Qualitative Analysis of Perceived Benefits

Inductive thematic analysis (Braun & Clarke, 2006) conducted on focus group and interview data with caregivers and interventionists identified the following themes regarding perceived benefits: (a) increased parental self-efficacy (‘Parents seem more self-confident, able to cope and handle the child’); (b) reduced parental stress and anxiety (‘Anxiety went down’, ‘I am less frustrated because I see that my child is improving’); (c) improvements in child’s behavior and communication in response to parent’s behavior (‘Some children communicate more, others are more regulated’; ‘My son has improved because I, as a parent, have improved’) and (d) connectedness in the dyad (‘Parents feel more able and children are more in contact with them’; ‘Children are more in contact, they communicate more… parents have learnt to wait and can now understand what the child is communicating’).

## Discussion

We examined the effectiveness of the novel WHO Caregiver Skills Training program (Salomone et al., 2019) implemented for the first time in public outpatient child neuropsychiatry settings in Italy, through a pilot RCT. In summary, we observed high levels of feasibility of delivery by clinicians, excellent acceptability to caregivers and favorable effects, 3 months post-intervention, on dyadic fluency of the caregiver/child interaction, child non-verbal communication, caregiver skills supportive of the interaction, self-efficacy and stress. The effect size of the intervention was large for parent skills and moderate for parenting self-efficacy. Smaller effects were found for flow of the interaction, child gestures and parenting stress.

The feasibility of delivery, as reflected in high fidelity of implementation, high attendance rates and good self-reported viability of delivery, combined with the high acceptability to the target beneficiaries, provide evidence in support of the scalability of the CST in public health services in Italy. The user-facing intervention materials (participant booklets), the contents covered during the sessions and the training methodology were found to be well received and in line with caregivers’ needs, confirming the qualitative analysis of post-intervention feedback (Salomone et al., 2021). With respect to feasibility assessment, it is noteworthy that the fidelity of implementation of CST strategies in interaction with children during the home visits, while overall acceptable, was lower for some interventionists (Salomone et al., 2021). This likely reflects the variety of previous professional experiences of the clinicians, including receiving training and being directly involved in delivering ASD-specific behavioral interventions, rather than their seniority. As discussed more extensively in Salomone et al. (2021), the uptake of CST intervention strategies by part of specialist professionals who nonetheless have received limited training in developmental/behavioral methods (Roll-Pettersson et al., 2020) presents with specific challenges.

The estimation of the blinded primary outcomes derived from the free play caregiver/child interaction showed that, although change scores are in the hypothesized direction indicating the effects of the intervention, there were no significant differences between arms in child’s joint engagement, availability to interact and autism symptom severity. We report, however, strong treatment effects on the blinded parent outcome: parents in the treatment arm significantly improved in their ability to support the interaction by scaffolding the child’s actions, following in on the child’s focus, and appropriately modulating affective communication to enhance the interaction. With respect to the scaffolding skills, it is noteworthy that high scaffolding scores in the JERI coding system require that the parent successfully guides the child’s behaviors in positive ways that are observable within the interaction; as such, high quality scaffolding extends beyond the mere application of the ‘correct’ support strategies on the part of the parent and captures the child’s response. There was also a significant improvement in the dyadic flow of the free play interaction, which captures the parent and child ‘connectedness’, or reciprocal interest in interacting and reciprocity of shared topic, ‘balance’ between partners in their contribution to the interaction, and overall ‘fluency’, or flow and lack of rigidity in the play or conversational turns.

These findings of change 3 months after the end of treatment are of particular relevance as they reflect the role of both partners in the success of the interaction, and as such indirectly highlight a significant improvement in both parent and child behavior, in spite of a lack of a significant treatment effect on the child’s primary outcomes. This pattern of findings suggests that a longer follow-up may be necessary to detect changes on joint engagement or that changes might occur on specific aspects of joint engagement that were not measured here, such as how often the child coordinates attention between the partner and objects or integrates language into joint engagement.

To study early effects of the intervention, we conducted the same analyses performed on change scores 3 months post intervention also on data taken immediately after the end of the treatment, as a secondary analysis. Results indicate that the intervention had no significant effect on the study outcomes except for the JERI Parent support of interaction skills, which significantly increased in the treatment group with respect to the TAU condition. Since at 3 months post-intervention both a larger significant change on the parent skills outcome and a significant improvement in the flow of the interaction were found, it can be postulated that change in dyadic outcomes 3 months post intervention may be driven by earlier occurring changes in parents’ actions. Parents may need more time to practice skills that lead to greater flow of the interaction and in turn, potentially, to increased time spent in joint engagement. This interpretation is in keeping with the significant treatment effect found for use of spontaneous gestures, a core impairment in ASD (Mishra et al., 2020): increased gesture production may be a first, critical indicator of improvement in early communication as it predicts language development (Mundy et al., 1987) and later socio-communicative skills (Riva et al., 2021). The post-intervention qualitative feedback provided by interventionists and parents, who reported that mastery of CST strategies required more time than anticipated and advocated for enhancing the intensity of the intervention with booster sessions (Salomone et al., 2021), supports this understanding. There is indeed evidence that parent skills supporting the interaction are pivotal to promote developmental outcomes and reduction in symptoms, e.g. parental synchrony mediated change in autism symptom outcome in the PACT trial (Pickles et al., 2015).

We also found a treatment effect on self-report parent outcomes, including the autism-specific parental self-efficacy (but not the general parenting self-efficacy) and parental stress measures, indicating that CST is effective for proximal, rather than distal (Sandbank et al., 2021) wellbeing outcomes. Similarly, there was no indication of change on the child adaptive behavior domain, as reported in other caregiver mediated trials in both high- (Pickles et al., 2016) and low- resource (Divan et al., 2012) settings, perhaps reflecting poor sensitivity to change of the Vineland measure within a limited time-frame for interventions addressing, but not exclusively targeting, the child’s daily living skills. Overall, the pattern of findings on blind observational measures and self-report questionnaires is mirrored by the qualitative evidence derived from focus groups with interventionists and caregivers.

There are several methodological strengths to the present study. Firstly, the sample size for this community-implemented caregiver mediated intervention evaluated through a randomized controlled design, the first of this kind conducted in public outpatient health settings in Italy, was relatively large – the median sample size in a recent review of interventions for preschoolers which included 48 studies was n = 49 (French & Kennedy, 2018). This, combined with the rigorous clinical characterization of participants through standardized assessments and the use of primary outcomes that were blind-rated, to a high level of reliability, allows for a better understanding of the effectiveness of the intervention in a community setting.

Nevertheless, the results should be interpreted in the context of some design features that may limit the interpretation of findings. First, while the CST program was developed to cater for a range of neurodevelopmental disorders and delays, recruitment was restricted to children with ASD to reduce the sample’s heterogeneity, which restricts the generalizability of our findings, particularly with respect to child outcomes. Similarly, as the CST was originally designed to be implemented by non-specialists, the relevance and applicability of some of our feasibility and acceptability findings may be limited to settings employing interventionists with similar levels of qualification and supervision.

A number of conclusions can be drawn from this pilot RCT of the CST in Italy. The CST was found to be acceptable and feasible. While balanced across groups, the sample was representative of the clinical population of children with ASD and as such characterized by high heterogeneity in clinical profiles, which indicates that our effectiveness trial could well be generalizable. We report treatment effects at family level outcomes, albeit proximal, which are important as they support the effective implementation of interventions in real-world contexts, maximizing the adoption and sustainability of caregiver-mediated interventions within community settings (Wainer et al., 2017). The pattern of change in parent, child and dyad outcomes suggest that strategies to increase the intensity of the intervention, such as the addition of booster group sessions and individual coaching to parents, may be critical to improve its effectiveness. Future research steps should examine treatment mediators and moderators to identify patterns of response to treatment by child and parent characteristics (Smith et al., 2007) and build on the present evidence of the theorized mechanism of intervention effect on proximal outcomes derived from caregiver/child interaction to examine intervention outcomes that may be less prone to correlated measurement error (Crank et al., 2021).
